# Clinical and laboratory features of SARS-CoV-2 variants across multiple rounds of pandemic waves in hospitalized children in an Iranian referral hospital

**DOI:** 10.1186/s12887-023-04042-w

**Published:** 2023-05-16

**Authors:** Shima Mahmoudi, Babak Pourakbari, Sepideh Benvari, Reihaneh Hosseinpour Sadeghi, Mohammad Reza Abdolsalehi, Mohammad Ali Shahbabaie, Fatemeh Jalali, Fatemeh Safari, Amene Navaeian, Setareh Mamishi

**Affiliations:** 1 Pediatric Infectious Disease Research Center, Tehran University of Medical Sciences, Tehran, Iran; 2 Biotechnology Centre, Silesian University of Technology, Gliwice, 44-100 Poland; 3 Department of Microbiology, Faculty of Medicine, Qazvin University of Medical Sciences, Qazvin, Iran; 4 Department of Infectious Diseases, Pediatrics Center of Excellence, Tehran University of Medical Sciences, Tehran, Iran

**Keywords:** SARS-CoV-2, Variants of concern, Clinical features

## Abstract

**Background:**

Since the onset of the COVID-19 pandemic, SARS-CoV-2 has evolved into independent new forms, variants of concern (VOCs). While epidemiological data showed increased transmissibility of VOCs, their impact on clinical outcomes is less clear. This study aimed to investigate the differences between the clinical and laboratory features of children infected with VOCs.

**Methods:**

This study included all cases with SARS-CoV-2-positive nasopharyngeal swabs obtained from patients referred to Children’s Medical Center (CMC), an Iranian referral hospital, between July 2021 and March 2022. The inclusion criteria for this study included all patients, regardless of age, who had a positive test anywhere in the hospital setting. Exclusion criteria for the study included those whose data was obtained from non-hospital outpatient settings, or referred from another hospital. The SARS-CoV-2 genome area encoding the S1 domain was amplified and sequenced. The type of variant in each sample was identified based on the mutations in the S1 gene. Demographic characteristics, clinical data, and laboratory findings were collected from the patient’s medical records.

**Results:**

This study included 87 pediatric cases with confirmed COVID-19, with a median age of 3.5 years (IQR: 1-8.12). Data from sequencing reveals the type of variants as 5 (5.7%) alpha, 53 (60.9%) Delta, and 29 (33.3%) Omicron. The incidence of seizure was higher in patients with Alpha and Omicron infection compared to the Delta group. A higher incidence of diarrhea was reported in Alpha-infected patients, and a higher risk of disease severity, distress, and myalgia was associated with Delta infection.

**Conclusion:**

Laboratory parameters did not mostly differ among the patients infected with Alpha, Delta, and Omicron. However, these variants may manifest different clinical features. Further studies with larger sample sizes are required to fully understand the clinical manifestations of each variant.

## Background

The coronavirus disease 2019 (COVID-19) is caused by severe acute respiratory syndrome coronavirus 2 (SARS-CoV-2), which was first reported in Wuhan city, China, in December 2019 [1]. As of May 2022, the pandemic had caused more than 510 million cases and 6 million deaths, making it one of the deadliest in history. In Iran, the first case of COVID-19 was identified in Qom city in February 2020. Since the first confirmed case, the number of cases has risen and resulted in more than 7 million cases infected and 140 thousand deaths [2].

COVID-19 can cause mild to severe illness; its clinical manifestations include fever, cough, shortness of breath, and in more severe cases, pneumonia, kidney failure, and eventually death. Apart from respiratory symptoms, gastrointestinal manifestations, including vomiting, diarrhea, constipation, and abdominal pain, are also common in COVID-19 patients [3, 4].

In children, SARS-CoV-2 infection has mostly resulted in mild or even asymptomatic infection [5–9]. However, a condition termed multisystem inflammatory syndrome in children (MIS-C) has emerged with the clinical manifestation of multi-organ involvement and occurred at a much-increased frequency compared to previous years [10–14].

The genomics analysis using next-generation sequencing (NGS) indicates that SARS-CoV-2 is an enveloped single-strand positive-sense RNA (+ ssRNA)- with a genome length of ∼ 30 kilobases encoding 9860 amino acids (aa) [15, 16]. The genome has 11 coding regions that can encode structural and non-structural proteins [17]. The main structural proteins include spike (s), envelope (E), membrane (M), and nucleocapsid (N). The S protein with a length of 1273 aa is one of the most important viral proteins that mediates receptor binding and virus entry. It has two major domains: S1 and S2. The S1 is responsible for receptor recognition and attachment, while S2 mediates cell fusion [18].

Genome sequence data have been extensively used to track the evolution of SARS-CoV-2 [19]. Most of the mutations of functional importance occur in S protein and can affect host tropism and viral transmission [20, 21]. Specific mutations in the S1 subunit of the S protein are considered genomic characteristics of variants of concern (VOCs) [22]. In October 2021, World Health Organization defines four VOCs: B.1.1.7 (Alpha) [23], B.1.351 (Beta) [24], P.1 (Gamma) [25], and B.1.617.2 (Delta) [26]. As of December 2021, a new VOC, B.1.1.529 (Omicron) variant, has been detected and has spread globally [27].

Each variant harbors several mutations. The Alpha-variant harbors N501Y and P681R mutations. The Beta-variant harbors these mutations with the addition of the E484K and K417N mutations. The Delta-variant has several spike mutations including T19R, G142D, E156G/Δ 157–158, L452R, T478K and D950N [28]. Omicron-variant harbors multitude of mutations (more than 30) in the spike gene, which increase the concerns of enhanced infectivity and immune escape ability [29].

Although there should be evidence of increased transmissibility, virulence, or immunity for a lineage to be classified as a VOC [30], the impact of the evolution on clinical outcomes, especially in children, is yet to be fully understood. Therefore, in this study, we analyzed the S1 domain sequencing data obtained from pediatric patients to investigate the frequency of VOCs and the relation between the variants and laboratory and clinical data in children.

## Materials and methods

### Patient selection

This study obtained ethical approval from Tehran University of Medical Sciences, Tehran, Iran (IR.TUMS.CHMC.REC.1400.092).

This study included all cases with SARS-CoV-2-positive nasopharyngeal swabs obtained from patients referred to Children’s Medical Center (CMC), an Iranian referral hospital, between July 2021 and March 2022. CMC is one of the most experienced sub-specialized hospitals in Iran, with nearly 20 specialty and sub-specialty wards and a monthly turnover rate of more than 35,000 outpatients and 2500 inpatients, offer high quality and specialized therapeutic services to neonates, infants and children throughout country.

Inclusion criteria for this study included all patients, regardless of age, who had a positive test anywhere in the hospital setting. Exclusion criteria for the study included those whose data was obtained from non-hospital outpatient settings, or referred from another hospital.

All cases were diagnosed with SARS-CoV-2 infection using a reverse transcription polymerase chain reaction (RT-PCR) assay of nasopharynx specimens, as previously described [10], or with MIS-C according to the Centers for Disease Control and Prevention (CDC) definition [31]. The following variables were collected: patient gender and age, symptoms at admission, comorbidities, severity of disease, laboratory parameters, ICU need, and mortality. All patients’ parents or guardians signed informed consent forms.

A severe COVID-19 diagnosis will require at least one of the following conditions: SpO2 93% (90% in premature infants), PaO2 60 mmHg, PaCO2 > 50 mmHg, a respiratory rate of 70/min (≤ 1 year) and 50/min (more than one year), or lung infiltrates > 50%; respiratory failure demanding respiratory support, septic shock development, or critical organ failure demanding intensive care unit (ICU) care [32]. The lab results were obtained from the patient’s medical records.

Laboratory tests including leukocyte, neutrophil, and lymphocyte counts, hemoglobin, platelet count, C-reactive protein (CRP), erythrocyte sedimentation rates (ESR), d-dimer, blood urea, serum creatinine, liver enzymes, fibrinogen, ferritin, lactate dehydrogenase, creatine phosphokinase, sodium, potassium, calcium, phosphorus, magnesium, albumin, procalcitonin, prothrombin time, partial thromboplastin time, and international normalized ratio, vitamin D, CK-MB, D-dimer, and troponin were assessed using the laboratory reference values.

All symptoms, including COVID-19 symptoms, were documented at the time of admission (e.g., fever, cough, conjunctivitis, shortness of breath, abdominal pain, vomiting or diarrhea, tachypnea, chest pain, headache, myalgia, rash, and seizure).

**Molecular reaction** After viral RNA extraction using High Pure Viral Nucleic Acid kit (Roche, Germany) according to the manufacturer’s instruction, and cDNA synthesis was accomplished.

using PrimeScript RT reagent kit (taKaRa, Japan) [33]. the cDNAs were subsequently used in routine PCR reactions, utilizing the specific primers targeting RBD domain (RBD_S_F: 5′- GGGCAAACTGGAAAGATTGCTGA-3′; RBD_S_R: 5′-TGTGTACAAAAACTGCCATATTGCA–3′). Amplification was performed in a mixture consisting of 2.5 µl of the 10X PCR buffer, 0.5 µL of 100 mM MgCl_2_, 2.5 units of *Taq* DNA polymerase, 0.5 µL of 50 µM dNTP, 0.5 µL of 10 pMol of each primer, 1 µl of cDNA (final concentration 2 ng/µL) and DNase-, RNase-free deionized water to a final volume of 25 µl. Cycling conditions were carried out as follows: initial denaturation at 95º C for 5 min, followed by 30 cycles including denaturation for 45 s at 95º C, annealing for 45 s at 60º C and extension for 45 s at 72º C and a single final extension at 72º C for 5 min. The PCR products were sent to be sequenced by the Sanger protocol (Bioneer, South Korea).

### Bioinformatics and statistical analysis

Full-length SARS-CoV-2 gene sequences were obtained from the NCBI GenBank database and the GISAID EpiFlu database (http://www.GISAID.org). This study screened and analyzed genome sequences with mutations in the S protein RBD. MAFFT version 7 online serve with default parameters (https://mafft.cbrc.jp/alignmeloadnt/server/) and BioEdit were used to align the sequences, and the Wuhan-Hu-1 (NC 045512) strain serving as a reference.

The sequencing result was analyzed as a FASTA file using the GISAID software algorithm. SPSS (Statistical Package for the Social Sciences) version 13.0 software was used for statistical analysis (SPSS Inc.). All categorical variables were represented numerically and as percentages. Means, standard deviations, medians, and interquartile ranges were used to describe normally and non-normally distributed data (IQR). To compare normally distributed, non-normally distributed continuous, and categorical variables, parametric tests (t-test and ANOVA), nonparametric tests (Mann–Whitney and Kruskal–Wallis), and the Pearson χ2 test (or Fisher exact test, when necessary) were used, respectively. Significance was considered at p < 0.05.

## Results

A total of 87 pediatric cases were included in the present study. The median age of the patients was 3.5 years (range: 8 days to 14 years, IQR: 1-8.12 years). Underlying conditions were found in 28 cases including cardiovascular diseases (n = 9), cerebral palsy (n = 3), esophageal atresia (n = 2); and hydrocephalus, autoimmune encephalitis, autoimmune hepatitis, talasemia major, inflammatory bowel disease, cystic fibrosis, acute lymphocytic leukemia, asthma, cirrhosis, seizure, diabetes, brain tumor, neurodevelopmental disorder, Glanzmann thrombasthenia (n = 1). The proportions of patients requiring ICU admission and respiratory support were 11.5% and 4.3%, respectively. The proportions of individuals with serious COVID-19 illness were 25.3%, and 8 cases (9.2%) had MIS-C. Remdesivir therapy for COVID-19 was reported in 14 patients (16.1%). In this study, the mortality rate was 2.3% (Table 1).

**Table 1:** Clinical characteristics of pediatric cases infected with Alpha, Delta, and Omicron variants

Characteristics		Variant				P value
		Alpha (N = 5) n, n/N (%)	Delta (N = 53) n, n/N (%)	Omicron (N = 29) n, n/N (%)	Total (N = 87) n, n/N (%)	
Sex	Male	2 (40)	35 (66)	8 (80)	45 (66.2)	0.303
	Female	3 (60)	18 (34)	2 (20%)	23 (33.8)	
Remdesivir		1/5 (20)	11/53 (20.8)	2/29 (6.9)	14/87 (16.1)	0.256
ICU admission		1/5 (20)	8/53 (15.1)	1/29 (3.4)	10/87 (11.5%)	0.237
MIS-C		1/5 (20)	5/53 (9.4)	2/29 (6.9)	8/87 (9.2)	0.642
Underlying disorders		2/5 (40)	18/53 (34)	8/29 (27.6)	28/87 (32.2)*	0.780
Severity of COVID-19		1/5 (20)	19/53 (35.8)	2/29 (6.9)	22/87 (25.3)	**0.015**
The need for respiratory support**		1/5 (20)	2/53 (3.8)	1/29 (3.4)	4/87 (4.3)	0.238
Invasive airway intubation		1/5 (20)	0/53 (0)	1/29 (3.4)	2/87 (2.3)	**0.015**
Death		0/5 (0)	1/53 (1.9)	1/29 (3.4)	2/87 (2.3)	0.849
Family history of COVID-19		2/5 (40)	41/53 (77.4)	17/29 (58.6)	60/87 (69)	0.076
**Signs and symptoms**						
Fever		3/5 (60)	47/52 (90.4)	23/28 (82.1)	73/85 (85.9)	0.138
Cough		1/5 (20)	29/51 (56.9)	10/28 (35.7)	40/84 (47.6)	0.088
Sore throat		0/5 (0)	2/51 (3.9)	0/28 (0)	2/84 (2.4)	0.515
Conjunctivitis		0/5 (0)	2/53 (3.8)	1/28 (3.6)	3/86 (3.5)	0.908
Tachypnea		1/5 (20)	13/53 (24.5)	4/28 (14.3)	18/86 (20.9)	0.559
Chest pain		0/5 (0)	4/53 (7.5)	2/28 (7.1)	6/86 (7)	0.818
Rhinorrhea		0/5 (0)	11/53 (20.8)	3/28 (10.7)	14/86 (16.3)	0.303
Vomiting		2/5 (40)	11/53 (20.8)	9/28 (32.1)	22/86 (25.6)	0.401
Headache		0/5 (0)	3/52 (5.8)	3/28 (10.7)	6/85 (7.1)	0.582
Abdominal pain		1/5 (20)	3/53 (5.7)	0/28 (0)	4/86 (4.7)	0.126
Diarrhea		2/5 (40)	3/53 (5.7)	5/28 (17.9)	10/86 (11.6)	**0.033**
Respiratory distress		1/3 (33.3)	15/53 (28.3)	2/28 (7.1)	18/85 (21.1)	**< 0.0001**
Myalgia		0/4 (0)	4/53 (7.5)	0/28 (0)	4/85 (4.7)	**0.001**
Rash		1/5 (20)	1/53 (1.9)	1/28 (3.6)	3/86 (3.5)	0.108
Seizure		2/5 (40)	4/53 (7.5)	7/27 (25.9)	13/85 (15.3)	**0.028**

ICU = Intensive care unit

MIS-C = Multisystem inflammatory syndrome in children

*Underlying conditions including cardiovascular diseases (n = 9), cerebral palsy (n = 3), esophageal atresia (n = 2); and hydrocephalus, autoimmune encephalitis, autoimmune hepatitis, talasemia major, inflammatory bowel disease, cystic fibrosis, acute lymphocytic leukemia, asthma, cirrhosis, seizure, diabetes, brain tumor, neurodevelopmental disorder, Glanzmann thrombasthenia (n = 1)

** including noninvasive ventilation (NIV) and invasive mechanical ventilation,

### Sequence analysis

Of 87 patients, 5 (5.7%) were determined to be infected with the Alpha variant based on the analysis of the S1 mutations. Also, 53 (60.9%) and 29 (33.3%) cases were infected with Delta and Omicron variants, respectively. Sequence alignment of spike RBD of SARS-CoV-2 variants showed that the alpha variant had 3 common mutations T716I, P681H and A570D, the last one was reported in all 5 patients. The delta type in this study had 4 common mutations namely D614G, T478K, L452R, and 9681R, and the vast majority (51 cases, 96.2%) had all the first three cases and 39 cases had the last mutation (73.6%). While the omicron type had 3 mutations N679K, E484K and Q498R, the first one was common in all 29 cases and the second in 25 cases (about 86%) and the last in about half of the patients with omicron (15 people, 51.7%) reported. It should be noted that all 87 investigated patients had the D614G mutation.

The median days of hospital stay were 5 days (range 1 to 46 days, IQR:3–6 day). The Delta variant showed a higher risk of disease severity compared to other variants (p value = 0.015). The proportions of patients requiring ICU admission during the Delta and Alpha period were higher, but the differences were not statistically significant. The main clinical symptoms include fever (85.9%) and cough (47.6%). Most of the clinical characteristics did not differ between the Alpha, Delta, and Omicron variant-infected patients, as detailed in Table 1.

Fever was common clinical symptoms in all peaks. Gastrointestinal symptoms, including diarrhea and vomiting were the highest symptoms in Alpha variant period.

Among all cases, 13 (15.3%) presented with seizures. The incidence of seizure was higher in patients with Alpha and Omicron infection compared to the Delta group (p value = 0.028). Myalgia was only found in Delta variant-infected patients (7.5%). Respiratory distress was significantly higher in those who infected with Delta variant (p < 0.0001).

Laboratory data did not differ between the variants, except for CRP and troponin levels (Table 2). Although a higher troponin level was found in the Omicron group, it was still in the normal range.

**Table 2:** Laboratory findings of pediatric cases infected with Alpha, Delta, and Omicron variants

Variable (normal range)	Variant				P value
	Alpha	Delta	Omicron	Total	
White blood cell count (4–10 × 10^9^/L)	7.68 ± 4.84	7.54 ± 3.42	8.63 ± 3.55	7.91 ± 3.54	0.409
Red blood cell count (3.5–5.5 × 10^9^/L)	4.09 ± 1.07	15.63 ± 60.03	4.49 ± 0.54	11.25 ± 47.00	0.561
Hemoglobin (10–16 g/dL)	10.86 ± 2.56	12.13 ± 1.37	12.32 ± 1.48	12.12 ± 1.50	0.131
Platelet count (150–450 × 10^9^/L)	241.00 ± 98.05	261.47 ± 94.42	268.90 ± 111.61	262.77 ± 99.67	0.840
Neutrophil count (2–7 × 10^9^/L)	4.76 ± 3.74	4.33 ± 3.47	5.02 ± 2.64	4.59 ± 3.21	0.650
Lymphocyte count (0.8-4.0 × 10^9^/L)	2.04 ± 1.73	2.41 ± 1.80	2.66 ± 2.33	2.47 ± 1.97	0.761
Blood urea nitrogen (5–20 mg/dL)	12.40 ± 12.38	12.99 ± 13.94	12.07 ± 7.66	12.65 ± 11.97	0.946
Creatine phosphokinase (24–172 U/L)	110.25 ± 83.58	173.31 ± 239.60	1329.75 ± 3911.56	416.61 ± 1825.64	0.148
Lactate dehydrogenase (5–746 U/L)	494.00 ± 50.41	558.97 ± 268.56	587.38 ± 428.05	561.43 ± 304.09	0.865
Calcium (8.5–10.2 mg/dL)	8.95 ± 1.32	19.23 ± 64.35	8.93 ± 0.88	15.17 ± 50.10	0.723
Phosphorus (2.8–4.5 mg/dL)	4.30 ± 1.59	4.49 ± 1.41	5.08 ± 1.05	4.68 ± 1.32	0.206
Sodium (135–145 mmol/L)	136.20 ± 4.32	135.00 ± 22.74	137.15 ± 2.73	135.76 ± 17.94	0.882
Potassium (3.7–5.9 mmol/L)	3.78 ± 0.68	4.32 ± 0.61	4.09 ± 0.51	4.21 ± 0.63	0.070
Prothrombin time (9.5–13.5 s)	18.38 ± 5.35	16.70 ± 6.34	16.03 ± 2.66	16.65 ± 5.60	0.759
Partial thromboplastin time (< 65 s (< 2 months), 30–45 s (> 2 months))	43.25 ± 12.18	38.81 ± 10.36	35.27 ± 6.37	38.26 ± 9.75	0.276
International normalized ratio	1.35 ± 0.39	1.25 ± 0.50	1.18 ± 0.21	1.24 ± 0.44	0.758
Erythrocyte sedimentation rate (0–10 mm/h)	17.50 ± 18.45	15.88 ± 15.13	11.25 ± 6.23	14.42 ± 13.07	0.286
Vitamin D (20–40 ng/mL)	25.50 ± 11.15	30.08 ± 8.95	24.50 ± 12.32	28.56 ± 9.80	0.379
CK-MB (5–25 IU/L)	21.00 ± 11.14	35.30 ± 29.14	400.18 ± 1036.94	107.42 ± 469.04	0.085
Troponin (0–0.3 ng/ml)	0.01 ± 0.01	0.10 ± 0.07	0.15 ± 0.05	0.10 ± 0.07	**0.044**
Fibrinogen (150–350 mg/dL)	242.75 ± 42.94	306.67 ± 99.81	266.58 ± 76.72	294.19 ± 94.24	0.229
C-reactive protein (< 6 mg/L)	4 (1.65–25.5)	4 (1.9–28.5)	1 (1–2)	2 (1–13)	**< 0.0001**
Magnesium (1.7–2.2 mg/dL)	2.15 (1.85–2.75)	2.2 (1.85–2.75)	2.1 (1.9–2.4)	2.1 (1.9–2.4)	0.27
Aspartate aminotransferase (10–40 U/L)	40 (24.5–888)	40 (24.5–888)	32 (22–41)	38 (29-47.7)	0.09
Alanine transaminase (10–40 U/L)	17.5 (15.2-209.5)	17.5 (15.2-209.5)	20 (13–22)	18.5 (16–25)	0.65
Ferritin (30–220 ng/mL)	85.5 (56.5–611)	91.5 (43.5–172)	66.5 (42–169)	87 (45.5–170)	0.75
D-dimer (< 500 ng/ml)	268.5 (136–5545)	353 (200–668)	500 (262–863)	357 (201–681)	0.7

## Discussion

Since the onset of the pandemic, SARS-CoV-2 has developed various mutations in different countries, some of which resulted in more virulent and treatment-resistant strains [34]. Mutations in S protein are of great interest since this protein is one of the main targets for drugs and vaccines against SARS-CoV-2. Therefore, continuous monitoring of S protein in each region can provide valuable data for disease prevention and treatment in that country [35]. In this study, we aim to: first, identify the type of the SARS-CoV-2 variant in children with COVID-19 based on the S1 mutations; second, investigate the relationship between the SARS-CoV-2 variant and the clinical features.

Based on the result of this study, most of the children (60.9%) were infected with the Delta variant. The second most dominant variant was Omicron (33.3%), and only a few (5.7%) patients had an Alpha variant infection. No patients were infected with Beta or Gamma variants. However, these data are specific to the time studied, which was between the Delta and Omicron surges in Iran. Among the mutations identified in this study, D614G mutation, which is considered to be a common global mutation, was reported in all samples. Also, in another study in India, it was observed in 81.66% of the sequenced genome [36]. Also, in the study of Dolci et al., a total of 32 children with SARS-CoV-2, half of whom were girls, with an average age of 1.4 years (range: 1 day to 13 years) were included in the study. Amino acid substitution D614G was detected in all isolated viral strains. One Delta and Omicron type was detected in 7 (21.8%) isolates and 1 (3.1%) case, respectively [37].

Our study shows that the laboratory data did not differ between the variants, except for troponin, which was higher in patients with Omicron infection. However, the mean level of troponin was still in the normal range. .

In this study, severe disease, distress, and myalgia were more frequent in Delta-infected patients. Ong et al. conducted a study on 829 patients infected with Alpha, Beta, and Delta variants. Their results showed that there is a possible correlation between the Delta infection and severe COVID-19, increased viral load, and prolonged viral shedding in respiratory samples [38]. In another study by Deng et al., it was shown that mild status is more prevalent with Omicron compared to Delta (50.0% vs. 22.1%, P value < 0.001) [39]. In addition, the result of a study by Wang et al. indicates that first-time infection with Omicron is associated with less severe symptoms compared to first-time infection with Delta [40].

Based on our results, children with an Alpha infection had a higher incidence of diarrhea. Jain et al. [41] compared the clinical symptoms of patients with MIS-C in Children during the first and second surges (original/Alpha) and third surge (Delta). Their results showed that children infected with original/Alpha had a higher incidence of intubation compared to Delta-infected children (16% vs. 0%, p value: 0.009), but gastrointestinal symptoms were not different between the two groups. The lack of difference in gastrointestinal symptoms might be due to the study design since the MIS-C patients mainly present gastrointestinal signs and symptoms [42].

In conclusion, our study enhances the current knowledge concerning the differences between VOCs in the case of signs and symptoms. There were not many differences between the Alpha, Delta, and Omicron variants in laboratory data. However, the analysis of clinical symptoms reveals that Delta is associated with the increased severity of illness, myalgia, and distress, while diarrhea was reported more in Alpha-infected patients. One limitation of our study is a small sample size which can cause missing the statistically significant differences in clinical presentation with VOCs. Further research with larger sample sizes is required to gain a full understanding of the differences in laboratory and clinical data between the VOCs.

## Data Availability

The data that support the findings of this study are available from the corresponding author upon reasonable request. All data is already released and that any accession numbers/web links are available (www.ncbi.nlm.nih.gov/genbank/), and that all web links and accessions allow access to public data. The datasets generated and/or analyzed during the current study are available in MAFFT version 7 online serve with default parameter (https://mafft.cbrc.jp/alignment/server/). Spike amino mutations were analyzed and calculated with BioAider V1.334 using Wuhan-Hu-1 (NC_045512) strain.

## List of Abbreviations

COVID-19: Coronavirus disease 19
SARS-CoV-2: Severe acute respiratory syndrome coronavirus 2
RT-PCR: Reverse Transcription polymerase chain reaction
MIS-C: Multisystem inflammatory syndrome in children
NGS: Next-generation sequencing
VOCs: Variants of concern
CRP: C-reactive protein
ESR: Erythrocyte sedimentation rates
